# John Barleycorn

**Published:** 1913-10

**Authors:** 


**John Barleycorn.**

Jack London's story in the *Saturday Evening Post* has made an impression, and not without reason—witness the following extract:

"It was my unmitigated good fortune, good luck, chance—that brought me through the fires of John Barleycorn. My life, my career, my joy in living, have not been destroyed. They have been scorched, it is true; but, like the survivors of forlorn hopes, they have by unthinkably miraculous ways come through the fight to marvel at the tally of the slain.

And like such a survivor of old red war, who cries out, 'Let there be no more war!' so I cry out, 'Let there be no more poison-fighting by our youths!' The way to stop war is to stop it—the way to stop drinking is to stop it. The way China stopped the general use of opium was by stopping the cultivation and importation of opium. The philosophers, priests and doctors of China could have preached themselves breathless against opium for a thousand years, and the use of opium, so long as opium was ever accessible and obtainable, would have continued unabated. We are so made—that is all.

We have with great success made a practice of not leaving arsenic and strychnine and typhoid and tuberculosis germs, lying round to destroy our children. Treat John Barleycorn the same way. Stop him. Don't let him lie 'round licensed and legal, to pounce upon our youth. Not of alcoholics or for alcoholics do I write, but for our youths, for those who possess no more than the adventure stings and the genial predispositions, the social man-impulses which are twisted all awry by our barbarian civilization that feeds them poison on all the corners. It is the healthy, normal boys now born or being born for whom I write.

It was for this reason more than any other, and more ardently than any other, that I rode down into the Valley of the Moon, all ajingle, and voted for equal suffrage. I voted that women might vote, because I knew that they, the wives and mothers of the race, would vote John Barleycorn out of existence and back into the historical limbo of our vanished customs of savagery. If I thus seem to cry out as one hurt, please remember that I have been sorely bruised, and that I do dislike the thought that any son or daughter of mine or yours should be similarly bruised.

The women are the true conservators of the race, the men are the wastrels, the adventure lovers and gamblers; and in the end it is by their women they are saved. About man's first experiment in chemistry was the making of alcohol, and down all the generations to this day man has continued to manufacture and drink it. And there has never been a day when the women have not resented man's use of alcohol, though they have never had the power to give weight to their resentment. The moment women get the vote in any community, the first thing they proceed to do is to close the saloons. In a thousand generations to come men of themselves will not close the saloons. As well expect victims of morphine to legislate it out of existence.

The women know. They have paid an incalculable price of sweat and tears for man's use of alcohol. Ever jealous for the race, they will legislate for the babes of boys yet to be born; and for the babes of girls too—for they must be the mothers, wives and sisters of these boys.

And it will be easy. The only ones that will be hurt will be the topers and seasoned drinkers of a single generation. I am one of these; and I make solemn assurance, based upon long traffic with John Barleycorn, that it won't hurt me very much to stop drinking when no one else drinks and when no drink is obtainable. On the other hand, the overwhelming proportion of young men are so normally non-alcoholic that, never having had access to alcohol, they will never miss it. They will know of the saloon only in the pages of history, and they will think of the saloon as a quaint old custom similar to bull baiting and the burning of witches."—*Maryland White Ribbon Herald.*

